# Preparation and Characterization of Micronized Artemisinin via a Rapid Expansion of Supercritical Solutions (RESS) Method

**DOI:** 10.3390/ijms13045060

**Published:** 2012-04-23

**Authors:** Huimin Yu, Xiuhua Zhao, Yuangang Zu, Xinjuan Zhang, Baishi Zu, Xiaonan Zhang

**Affiliations:** 1 Chinese Medicine Department, the Second Affiliated Hospital of Harbin Medical University, Harbin 150086, China; E-Mail: huimin1973@126.com; 2 Key Laboratory of Forest Plant Ecology, Ministry of Education, Northeast Forestry University, Harbin 150040, China; E-Mails: nefuzhangxinjuan@163.com (Xin.Z.); zbs616@126.com (B.Z.); zyzolan@yahoo.cn (Xia.Z.)

**Keywords:** RESS, supercritical fluids, micronization, artemisinin

## Abstract

The particle sizes of pharmaceutical substances are important for their bioavailability. Bioavailability can be improved by reducing the particle size of the drug. In this study, artemisinin was micronized by the rapid expansion of supercritical solutions (RESS). The particle size of the unprocessed white needle-like artemisinin particles was 30 to 1200 μm. The optimum micronization conditions are determined as follows: extraction temperature of 62 °C, extraction pressure of 25 MPa, precipitation temperature 45 °C and nozzle diameter of 1000 μm. Under the optimum conditions, micronized artemisinin with a (mean particle size) MPS of 550 nm is obtained. By analysis of variance (ANOVA), extraction temperature and pressure have significant effects on the MPS of the micronized artemisinin. The particle size of micronized artemisinin decreased with increasing extraction temperature and pressure. Moreover, the SEM, LC-MS, FTIR, DSC and XRD allowed the comparison between the crystalline initial state and the micronization particles obtained after the RESS process. The results showed that RESS process has not induced degradation of artemisinin and that processed artemisinin particles have lower crystallinity and melting point. The bulk density of artemisinin was determined before and after RESS process and the obtained results showed that it passes from an initial density of 0.554 to 0.128 g·cm^−3^ after the processing. The decrease in bulk density of the micronized powder can increase the liquidity of drug particles when they are applied for medicinal preparations. These results suggest micronized powder of artemisinin can be of great potential in drug delivery systems.

## 1. Introduction

**Figure 1:**
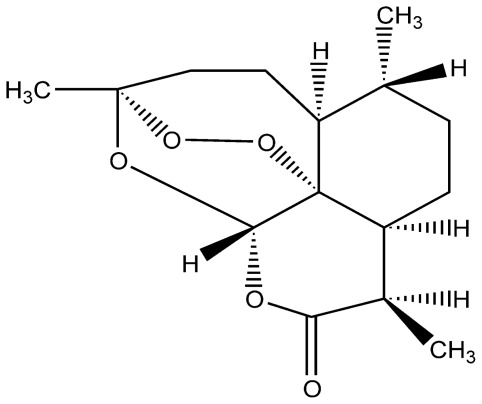
The chemical structure of artemisinin (the molecular weight is 282.34).

Artemisinin (chemical structure: Figure 1) is a sesquiterpene lactone with an endoperoxide function. It was first isolated from the Chinese traditional herb—*Artemisia annua* L. and its structure was first confirmed by Chinese scientists in the 1970s [1]. Artemisinin and its derivatives or analogues are currently regarded as the most promising weapons against multidrug-resistant malaria [2]. Its unique 1,2,4-trioxane structure is entirely incompatible with the traditional antimalarial structure-activity theory, which attracted the interest of many researchers [3,4]. However, artemisinin cannot be made into an injection due to its poor water solubility [5] and its very low oral bioavailability. Therefore, scientists remodel the molecular structure of artemisinin, to synthesize a series of derivatives, such as dihydroartemisinin, artemethe, arteether and artesunat and so on [6–8]. Although these derivatives can improve the solubility to a certain point, preparation techniques are difficult and costly. All of these limited the clinical application of artemisinin [9,10].

The bioavailability of pharmaceuticals presented in a solid formulation strongly depends on the size, particle size distribution and morphology of the particles [11]. Due to this, there is an increasing interest in the development of efficient micronization technologies. Different techniques have been applied for this purpose, including spray-drying, freeze-drying, liquid antisolvent crystallization or milling processes [12,13]. These technologies present several disadvantages, such as the production of coarse particles with broad particle size distribution, the degradation of the product due to mechanical or thermal stresses, or the contamination of the particles with organic solvents or other toxic substances. For this reason, different alternative precipitation methods are being investigated.

Rapid expansion of supercritical solutions (RESS) is a new technology that has developed in recent years [14–22]. In the RESS technique, the solute is first solubilized in a supercritical fluid and the supercritical solution is expanded through a fine-diameter nozzle. When a supercritical solution containing a dissolved solute is rapidly expanded across a micro-orifice, the solvent density dramatically decreases, leading to precipitation of the solute form the solvent [23,24]. The high supersaturation ratios and the homogeneous conditions attained due to the rapid expansion of a highly compressible supercritical mixture are the distinguishing features of the RESS process [25]. The RESS process can produce particles with submicroscopic size with narrow size distribution. The greatest advantage of the RESS process is the condition is mild and green, and especially it can be well used in the materials which are temperature-sensitive and have strong biological activity. In this study, artemisinin was micronized by RESS process with the purpose to improve the water solubility and bioavailability of artemisinin. The characterization of the artemisinin particles was carried out using SEM, LC-MS, FTIR, DSC and XRD.

## 2. Results and Discussion

### 2.1. Optimization of RESS-SC Micronization

The assignment of the experiment and the collected data for MPS of micronized artemisinin is shown in Table 1. The results showed that access to the largest of artemisinin micronization powder size was 2100 nm, the minimum diameter of 620 nm. According to the *R* value we can see that the influence to the MPS of micronized artemisinin decreases in the order: A > B > C > D, the best operating conditions is A_4_B_4_C_3_D_4_ (62 °C, 25 MPa, 45 °C and 1000 μm). Through a confirmatory test, smaller micronized artemisinin was obtained, with a minimum diameter of 550 nm. The yield of micronized artemisinin was about 86.2%.

**Figure 2:**
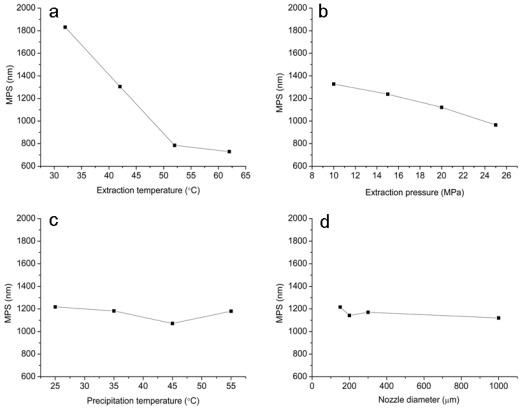
The effect of each parameter on the MPS of micronized artemisinin. (**a**) Extraction temperature; (**b**) Extraction pressure; (**c**) Precipitation temperature and (**d**) Nozzle diameter.

The relationships between the MPS of micronized artemisinin and process parameters are shown in Figure 2. As seen in Figure 2, the MPS of micronized artemisinin decreased as the extraction temperature increased from 32 to 62 °C and extraction pressure increased from 10 to 25 MPa, respectively. However, precipitation temperature and nozzle diameter within their given operational range have no significant influences on the MPS. Solution saturation has a significant effect on the crystallization of the solute, the greater degree of supersaturation, the smaller particle size of the crystal precipitation. It can be known from the best operating conditions, the smaller powder particle size had been obtained in the condition of high temperature and pressure. This is due to the increase of temperature and pressure that led to the rapid expansion of supercritical fluid and formed a very high saturation, and the solute formed of tiny crystalline nuclei in an instant.

The data are analyzed using Design Expert 7.0 software for evaluating the effect of each parameter on the optimization criteria. Table 2 lists the data of the ANOVA table of this experiment. The ANONA analysis revealed that each of the four factors exerted influence on the MPS of micronized artemisinin in the selected ranges, among which extraction temperature and pressure were identified as the most important determinant based on ANOVA with 95% confidence. Moreover, the particle size of micronized artemisinin decreased with increasing extraction temperature and pressure. The increasing solubility of artemisinin in SC-CO_2_ was determined as temperature or pressure increase [26]. The smaller particle size was obtained due to higher supersaturation caused by the higher solubility of artemisinin.

### 2.2. Particle Morphology

**Figure 3:**
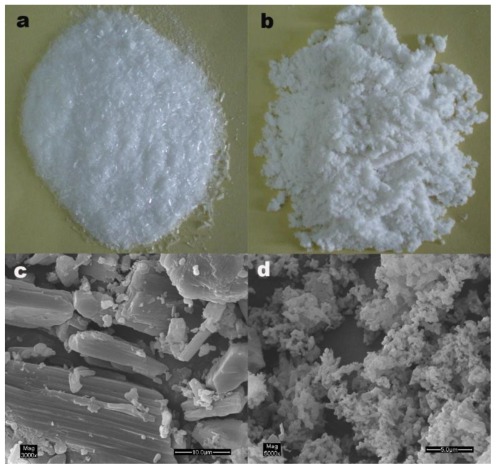
The particle morphology of unprocessed and processed artemisinin. (**a**) photograph of unprocessed artemisinin; (**b**) photograph of processed artemisinin; (**c**) SEM image of unprocessed artemisinin; (**d**) SEM image of processed artemisinin.

Particle morphology and particle size were defined on a visual basis. The photograph of the conventionally crystallized unprocessed drug shows narrow needle-like crystals (Figure 3a). It is visible from Figure 3c that unprocessed artemisinin particles are irregular needle-shaped crystals, ranging in length from 30 μm to 1200 μm. Processed artemisinin by RESS is a loose white powder RESS (Figure 3b). The SEM image shows that processed artemisinin is lamelliform particles, ranging in length from 400 nm to 850 nm (Figure 3d). A tendency to form aggregates is noted, this is because of smaller particle size and surface activity of the high reunion. It should be mentioned that these aggregates are completely separated when the powder is suspended in water.

### 2.3. FTIR Analysis

**Figure 4:**
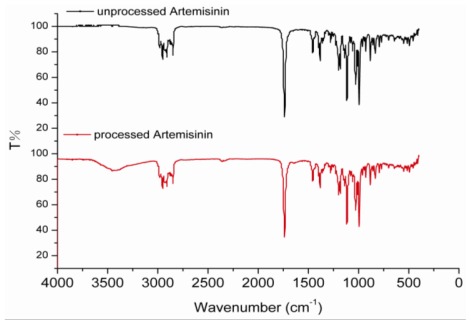
Comparison of artemisinin FTIR spectra before and after RESS processing.

The FTIR spectra of unprocessed and processed artemisinin were taken to obtain information on the change of chemical structure after RESS processing presented in Figure 4. It can be seen from Figure 4 that FTIR spectra between unprocessed and processed artemisinin do not show any significant differences. The assignment of bands are as follows: 995, 928 and 833 cm^−1^ (C-C stretching vibrations), 1027 cm^−1^ and 1012 cm^−1^ (-C-O-stretching vibrations), 1117 cm^−1^ (-O- stretching vibrations), 1384 cm^−1^ (-CH_3_ stretching vibrations), 1456 cm^−1^ (-CH_2_ Bending vibrations), 1738 cm^−1^ (C=O stretching vibrations), 2953 cm^−1^ and 2980 cm^−1^ (-CH_2_ stretching vibrations).

### 2.4. LC-MS Analysis

**Figure 5:**
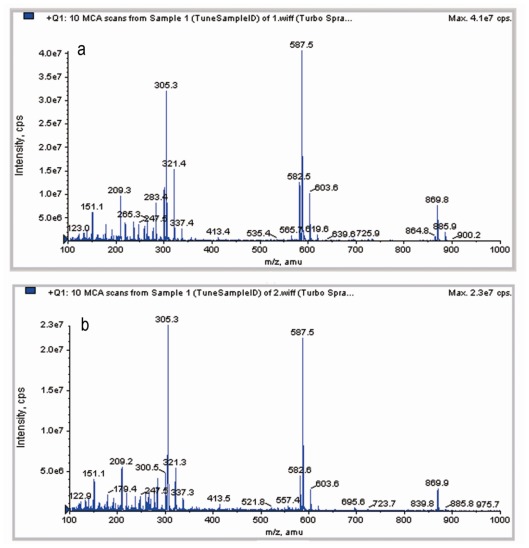
LC-MS analysis of artemisinin before and after RESS processing. (**a**) unprocessed artemisinin; (**b**) processed artemisinin.

The full scan mass spectra of unprocessed and processed artemisinin after direct injection in mobile phase are presented in Figure 5. As seen from Figure 5, no modification occurred in molecular weight. The protonated molecule was detected at *m/z* 283.34 for [M + H]^+^. This mass agrees with the published structure C_15_H_22_O_5_ of artemisinin. The two forms of artemisinin exhibited the same molecular weight (282.34). Therefore, the RESS process has not induced degradation of artemisinin.

### 2.5. DSC Analysis

**Figure 6:**
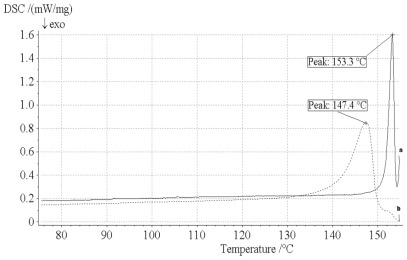
DSC analysis of before and after RESS processing. (**a**) Unprocessed artemisinin; (**b**) processed artemisinin.

The obtained particles were characterized by DSC (Figure 6). The melting point of unprocessed artemisinin is 153.3 °C whereas RESS-SC processed artemisinin has a melting point of 147.4 °C. The melting point of processed artemisinin was about 5.9 °C less than that of unprocessed artemisinin. Figure 6 shows also the heat flow with temperature plot of unprocessed and processed artemisinin particles. The decrease of heat flow for processed artemisinin can be attributed to the lowering of the crystallinity after RESS-SC processing.

### 2.6. X-ray Analysis

**Figure 7:**
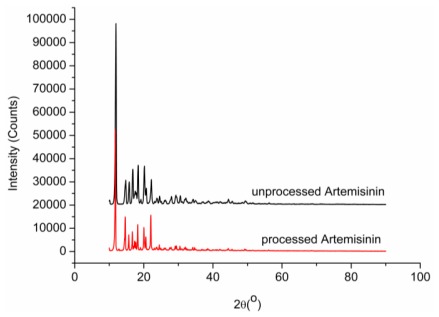
Comparison of artemisinin XRD traces before and after RESS processing.

Figure 7 shows the XRD results for processed and unprocessed artemisinin particles. Though the peaks are at the same angles (2θ = 11.94°, 18.36°, 20.14°), the intensity of the peaks are lower for RESS processed particles. It can be seen from Figure 7 that the initial state of unprocessed artemisinin is crystalline. The crystalline structure is partly retained because the main peaks are still present in the processed artemisinin trace. Lower intensity can be attributed to the lowering of crystallinity of the particles after RESS processing. Less crystalline and smaller drug particles are higher in the dissolution rate or bioavailability than crystals and, thus, the therapeutic action is obtained in shorter times [27]. Moreover, lower crystallinity materials (with partial crystalline structure) can be more stable in time than amorphous ones. The effect of the storage time on the morphology and the crystalline structure of artemisinin particles will be assessed in a future work.

### 2.7. Bulk Density

The results of bulk density determination obtained are presented in Table 3. The results show that bulk density of RESS-SC processed artemisinin is significantly less than unprocessed artemisinin. Formation of micronization with smaller particle size could be the reason for the lowering of intensity. This can increase the liquidity of drug particles when they are applied for medicinal preparations.

## 3. Experimental Section

### 3.1. Materials

Artemisinin was obtained from Xian Sino-herb Biotechnology Co., Ltd. (Shanxi, China). It was extracted from *Artemisia annua* L., and then purified. It is a colorless needle crystal and the purity of mass fraction was above 98.5%. Further purification was not performed. High purity CO_2_ (99.99% pure) was purchased from Liming gas company of Harbin (Heilongjiang, China).

### 3.2. RESS Apparatus

**Figure 8:**
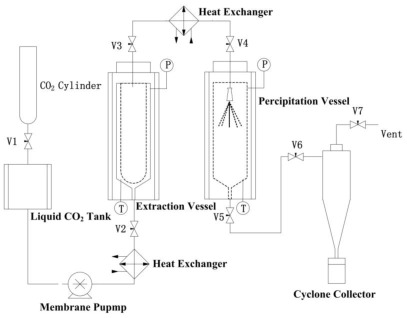
Schematic diagram of the rapid expansion of supercritical solutions (RESS) apparatus.

The schematic of RESS-SC process is shown in Figure 8. The apparatus consists of extraction chamber and precipitation chamber. In each experiment, 1000 mL stainless steel extraction column was used and 0.2 μm spare frits filters were placed at both ends for distribution of supercritical CO_2_. The purified gaseous CO_2_ is liquefied and subcooled in a liquid CO_2_ tank, and then compressed to the desired pressure with a high-pressure syringe pump. The preheated CO_2_ entered the extraction unit using a heat exchanger and formed a supercritical solution. The extraction column with material was placed in the temperature-controlled extraction chamber and equilibrated to the pre-set extraction temperature. The solvent (CO_2_) was delivered to the system at the extraction pressure by high-pressure syringe pumps and entered the extraction column. Subsequently, the supercritical solution with a mass flow of 6.0 kg/h was expanded rapidly through a nozzle into the precipitation chamber at atmospheric pressure. The CO_2_ mass flow through the nozzle is measured with a mass flow meter. The crystals produced were collected by a cyclone collector that was connected to precipitation chamber. The experiments were carried out in batch mode. In each experiment the system was given 30 min to reach steady state condition and then the supercritical fluid was expanded for 10 min in the precipitation vessel.

### 3.3. Optimization of RESS-SC Micronization

Artemisinin was put in the form of powder in the extraction chamber. Then artemisinin was dissolved in supercritical CO_2_ fluid. The artemisinin/CO_2_ solution was sprayed rapidly through a nozzle into precipitation chamber from a desired pressure to atmospheric pressure. The parameters affecting particle size include extraction pressure, extraction temperature, nozzle diameter and precipitation temperature. According to solubility of artemisinin in supercritical carbon dioxide [26], an orthogonal design OA_16_ (4)^5^ was selected for optimization of operating condition of artemisinin micronization by the RESS process. As indicated in Table 4, the RESS experiment is carried out with 4 factors and 4 levels, namely extraction temperature (32, 42, 52, 62 °C), extraction pressure (10, 15, 20, 25 MPa), precipitation temperature (25, 35, 45, 55 °C), and nozzle diameter (150, 200, 300, 1000 μm). The range of each factor level is based on the results of preliminary experiments. The MPS (nm) of micronized artemisinin is the dependent variable. The data are analyzed using the Design Expert 7.0 software (Stat-Ease, Inc.: Minneapolis, MN, USA).

### 3.4. Analytical Methods

#### 3.4.1. Observation of Particle Morphology

A SEM (Quanta 200, FEI Company, Eindhoven, The Netherlands) was used to observe the shape and measure the mean particle diameter of the unprocessed and processed artemisinin.

#### 3.4.2. FTIR Analysis

The unprocessed and processed artemisinin were diluted with KBr mixing powder at 1% and pressed to obtain self-supporting disks, separately. The FTIR spectrum was obtained by MAGNA-IR560 E.S.P (Nicolet, Madison, WI, USA) and recorded in the wave number range of 4000–500 cm^−1^ at a resolution of 4 cm^−1^.

#### 3.4.3. LC-MS Analysis

The unprocessed and processed artemisinin were dissolved separately in methanol. High performance liquid chromatography-mass spectrometry (LC-MS) was obtained by analyst 1.4 of AB API 3000 (Foster City, CA, USA). The mass spectrometer was operated in positive ion mode.

#### 3.4.4. DSC Analysis

Thermal analysis was carried out using DSC (TA instruments, model DSC 204) for processed and unprocessed artemisinin particles. Analysis was performed for 5.0 mg samples at a temperature heating rate of 5 °C/min and a temperature range of 20–200 °C.

#### 3.4.5. X-ray Diffraction

X-ray diffraction patterns were collected in transmission using an X-ray diffractometer with a rotating anode (Xpert-Pro, Philips, Almelo, The Netherlands) with Cu Kα_1_ radiation generated at 30 mA and 50 kV. The powders of unprocessed and processed artemisinin were filled to same depth inside the sample holder by leveling with spatula and scanning rate (4°/min) was same for all XRD analysis.

#### 3.4.6. Bulk Density

Bulk density of the powders was determined using the method described in the United States Pharmacopoeia XXVII [28]. Pass a quantity of powder sufficient to complete the test through a 1.0 mm sieve. Carefully scrape the excess powder from the top of the vessel (volume = *v*_0_). The mass (*m*_0_) of the powder was determined to the nearest 0.1 percent by subtraction of the previously determined mass of the empty measuring vessel. The bulk density (g/mL) was calculated by the formula *m*_0_/*v*_0_ and recorded the average of three determinations using three different powder samples.

## 4. Conclusions

In this study, artemisinin was micronized by the rapid expansion of supercritical fluids (RESS). Micronization particles of artemisinin with a MPS of 550 nm are obtained (at extraction temperature of 62 °C, extraction pressure of 25 MPa, precipitation temperature of 45 °C and nozzle diameter of 1000 μm). The particle size of micronized artemisinin decreased with increasing extraction temperature and pressure. The RESS process did not induce the degradation of artemisinin and the processed artemisinin particles have lower crystallinity and melting point. The bulk density of artemisinin was determined before and after RESS process, and the obtained results showed that it passes from an initial density of 0.554 to 0.128 g cm^−3^ after the processing. The decrease in bulk density of the micronized powder can increase the liquidity of drug particles when they are applied for medicinal preparations. This is due to the uniform size and good surface flatness of micronized artemisinin powder. These results suggest that micronized powder of artemisinin can be great potential in drug delivery systems.

## Figures and Tables

**Table 1:** Experimental conditions and results for the artemisinin RESS processes.

Trial No.	Extraction Temperature (°C)	Extraction Pressure (MPa)	Precipitation Temperature (°C)	Nozzle Diameter (μm)	MPS (nm)
1	1(32)	1(10)	1(25)	1(150)	2100
2	2(42)	1(10)	2(35)	2(200)	1930
3	3(52)	1(10)	3(45)	3(300)	1725
4	4(62)	1(10)	4(55)	4(1000)	1571
5	1(32)	2(15)	3(45)	2(200)	1459
6	2(42)	2(15)	4(55)	1(150)	1411
7	3(52)	2(15)	1(25)	4(1000)	1358
8	4(62)	2(15)	2(35)	3(300)	992
9	1(32)	3(20)	4(55)	3(300)	840
10	2(42)	3(20)	3(45)	4(1000)	880
11	3(52)	3(20)	2(35)	1(150)	740
12	4(62)	3(20)	1(25)	2(200)	680
13	1(32)	4(25)	2(35)	4(1000)	910
14	2(42)	4(25)	1(25)	3(300)	730
15	3(52)	4(25)	4(55)	2(200)	660
16	4(62)	4(25)	3(45)	1(150)	620
*K_1_a*	1831.5	1327.25	1217.75	1217.0	
*K_2_*	1305.0	1237.75	1182.25	1143.0	
*K_3_*	785.0	1120.75	1071.75	1171.0	
*K_4_*	730.0	965.75	1179.75	1120.5	
*R_b_*	1101.5	361.5	146.0	96.5	
Optimal level	A_4_	B_4_	C_3_	D_4_	

a *K_i_* *^A^* = ∑(mean particle size at A_i_)*/*4, the mean values of mean particle size for a certain factor at each level with standard deviation;

b *R_i_* *^A^* = max{*K_i_* *^A^*} − min{*K_i_* *^A^*}.

**Table 2:** ANONA analysis of four parameters for RESS micronization of artemisinin.

Source	Sum of Squares (SS)	Degrees of Freedom (df)	*F*-Ratio	*F*_0.05_	Type of Effect
A	3189716.75	3	334.045	9.280	Significant
B	293032.75	3	30.688	9.280	Significant
C	47900.75	3	5.016	9.280	
D	20744.75	3	2.173	9.280	
Error	9548.75	3	22075		

**Table 3:** The comparation of bulk density between unprocessed and processed artemisinin.

Artemisinin	Quality (g)	Volume (mL)	Density (g/mL)
unprocessed	2.77	5	0.554
processed	0.64	5	0.128

**Table 4:** The factors and levels of the orthogonal array design.

Factors	(A) Extraction Temperature	(B) Extraction Pressure	(C) Precipitation Temperature	(D) Nozzle Diameter
Levels	(°C)	(MPa)	(°C)	(μm)
1	32	10	25	150
2	42	15	35	200
3	52	20	45	300
4	62	25	55	1000
